# Comparative Analysis of the Major Chemical Constituents in *Salvia miltiorrhiza* Roots, Stems, Leaves and Flowers during Different Growth Periods by UPLC-TQ-MS/MS and HPLC-ELSD Methods

**DOI:** 10.3390/molecules22050771

**Published:** 2017-05-10

**Authors:** Huiting Zeng, Shulan Su, Xiang Xiang, Xiuxiu Sha, Zhenhua Zhu, Yanyan Wang, Sheng Guo, Hui Yan, Dawei Qian, Jinao Duan

**Affiliations:** 1 Jiangsu Collaborative Innovation Center of Chinese Medicinal Resources Industrialization, National and Local Collaborative Engineering Center of Chinese Medicinal Resources Industrialization and Formulae Innovative Medicine, and Key Laboratory of Chinese Medicinal Resources Recycling Utilization, State Administration of Traditional Chinese Medicine, Nanjing University of Chinese Medicine, Nanjing 210023, China; Zenght1991@163.com (H.Z.); xx5326@126.com (X.X.); shaxiu901128@yeah.net (X.S.); 04040416@163.com (Z.Z.); wyy8127@163.com (Y.W.); gsh916@163.com (S.G.); glory-yan@163.com (H.Y.); qiandwnj@126.com (D.Q.); 2 Department of Traditional Chinese Medicine, Jiangxi Province Academy of Traditional Chinese Medicine, Nanchang 330046, China

**Keywords:** aerial parts, distribution, dynamic changes, harvest time, *S. miltiorrhiza* Bunge

## Abstract

*Salvia miltiorrhiza* is a traditional Chinese herbal medicine containing multiple components that contribute to its notable bioactivities. This article investigated the distribution and dynamic changes of chemical constituents in various parts of *S. miltiorrhiza* from different growth periods. An ultra-high performance liquid chromatography-triple quadrupole mass spectrometer (UPLC-TQ-MS/MS) and high-performance liquid chromatography coupled with evaporative light scattering detector (HPLC-ELSD) methods were developed for accurate determination of 24 compounds (including phenolic acids, flavonoids, triterpenes, and saccharides) in *S. miltiorrhiza*. The established methods were validated with good linearity, precision, repeatability, stability, and recovery. Results indicated that there were category and quantity discrepancies in different parts of the plant, for the roots mainly contained salvianolic acids and tanshinones, and most of the saccharides are stachyose. In the aerial parts, salvianolic acids, flavonoids, and triterpenes, except the tanshinones, were detected, and the saccharides were mainly monosaccharides. Dynamic accumulation analysis suggested the proper harvest time for *S. miltiorrhiza* Bunge was the seedling stage in spring, and for the aerial parts was July to August. This study provided valuable information for the development and utilization value of the aerial parts of *S. miltiorrhiza* and was useful for determining the optimal harvest time of the plant.

## 1. Introduction

**Figure 1:**
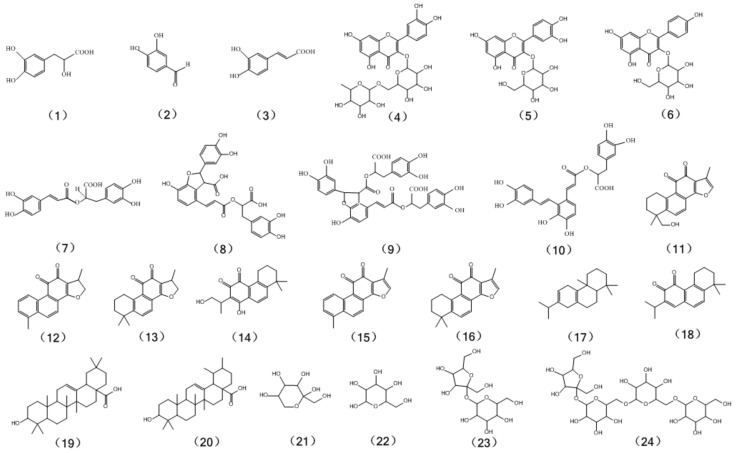
Chemical structures of 24 reference compounds in *S. miltiorrhiza*.

*Salvia miltiorrhiza* (SM), a kind of perennial herb that derived from *Salvia* genus of the Labiatae family, the dry roots and rhizomes are officially listed in the Chinese Pharmacopoeia [1,2], and it is highly valued and one of the most widely used traditional Chinese medicines (TCM) by virtue of its function to “promote blood circulation and remove blood stasis” that was firstly recorded in *Shennong's Classic of Materia Medica* (200–300 AD, Han Dynasty). The remarkable effects of *S. miltiorrhiza* on treatment of complicated cardiovascular diseases and cerebrovascular diseases have been reported during the past decades [3,4,5]. Additionally, numerous studies have demonstrated that *S. miltiorrhiza* possesses a wide range of pharmacological effects, including anti-oxidative, myocardial infarction, anti-fibrotic [6], anti-inflammatory [7], anti-hypertension [8] and anti-neoplastic [9] effects. Studies show that the efficacious and reliable biological activities of *S. miltiorrhiza*, primarily due to its hundreds of chemical components, which were roughly classified into hydrophilic salvianolic acids, include danshensu (**1**), protocatechuic aldehyde (**2**), caffeic acid (**3**), rosmarinic acid (**7**), lithospermic acid (**8**), salvianolic acid B (**9**), salvianolic acid A (**10**), etc. and diterpenoid tanshinones comprise of tanshinone IIB (**11**), dihydrotanshinone I (**12**), cryptotanshinone (**13**), neocryptotanshione (**14**), tanshinone I (**15**), tanshinone IIA (**16**), miltiradiene (**17**), miltirone (**18**), etc. [10,11,12]. The chemical structures of these reference compounds are shown in Figure 1.

On account of the important medicinal value, *S. miltiorrhiza*, and its relevant preparations, are now drawing worldwide attention with a series of products, such as Danshen injection, Fufang Danshen tablets, tanshinone capsules [13,14], and the Fufang Danshen Dripping Pill, whose phase III clinical study were completed in 2016 [15]. It is estimated that the annual demand of *S. miltiorrhiza* was about 5000–7000 tons in recent decades, and that the yield of *S. miltiorrhiza* based on natural resources was insufficient and gradually replaced by artificial cultivation, hence, giving rise to the expansion of planting areas and an increase of production. However, as is well known, the aerial parts of *S. miltiorrhiza*, whose biomass accounts for about 67% of the whole plant, was discarded as waste during the root and rhizome harvest, which results in a significant waste of resources and ecological environmental pollution [16]. Therefore, supposing that the aerial parts of *S. miltiorrhiza* (including the stems, leaves, and flowers) as non-traditional medicinal parts could be fully used, we can make the best of *S. miltiorrhiza* resources [17].

In recent years, studies about the aerial parts of *S. miltiorrhiza* have attracted increasing attention. To the best of our knowledge, the aerial parts of the plant contain abundant biologically-active ingredients [18], and its medicinal value has been cited earlier in the *Medical Compliance* of the Qing Dynasty for “promoting blood circulation and removing blood stasis”. Modern studies also showed that the aerial parts of *S. miltiorrhiza* possess biological activities, such as anti-virus, anti-tumor, anti-inflammation, anti-oxidation, promoting blood circulation and removing blood stasis, anti-atherogenic properties, and so on, and it can be used for the treatment of thrombosis, cardiovascular diseases, for instance for coronary heart disease, and diabetes glucose metabolism disorders [19,20,21,22,23]. Hitherto, the chemical constituents of the aerial parts of *S. miltiorrhiza* have been discovered and isolated gradually, primarily for the phenolic acids, flavonoids (mainly for rutin (**4**), isoquercitrin (**5**), astragalin (**6**), etc.), triterpenes (mainly for oleanolic acid (**19**) and ursolic acid (**20**)), and saccharides (such as fructose (**21**), glucose (**22**), sucrose (**23**), stachyose (**24**), and so on) [24,25,26]. The chemical structures of these reference compounds are shown in Figure 1. In a word, it is worthwhile to explore, develop and utilize the aerial parts of the plant.

Until now, there have been reports concerning the growth pattern and optimal collecting period of *S. miltiorrhiza* Bge. [27,28]. Nevertheless, there are no published reports about the distribution and dynamic accumulation of various parts of *S. miltiorrhiza* (roots, stems, leaves, and flowers). In the present study, on the basis of the preliminary experiments in our laboratory [29,30], samples from different parts of *S. miltiorrhiza* were collected from 2015 to 2016, the sensitive and fast ultra-high performance liquid chromatography-triple quadrupole mass spectrometer (UPLC-TQ-MS/MS) method was developed for simultaneous quantitation of 20 components (seven salvianolic acids, three flavonoids, eight tanshinones, and two triterpenes) in *S. miltiorrhiza* for the first time, which dramatically simplified the complicated chromatographic separation and identification for multiple components of this plant, and the high-performance liquid chromatography coupled with an evaporative light scattering detector (HPLC-ELSD) method was established for the detection of four saccharides. As a result, our study was attempted to provide a constructive approach for analyzing the changes of these chemical components in separate parts of *S. miltiorrhiza* at different growth periods. Furthermore, it provided the theoretical basis and scientific evidence for the development and utilization of traditional and non-traditional medicinal parts of *S. miltiorrhiza* resources. Meanwhile, the established and applied methods in this paper may be useful to the readers, and especially the newcomers, in the research area of *S. miltiorrhiza* resources.

## 2. Results and Discussion

### 2.1. Optimization of Mass Spectrometry and Chromatographic Conditions

In this study, in order to obtain the best mass spectrometry conditions, standard solutions of all of the analytes were tested separately in direct infusion mode by the full-scan MS method in both positive and negative ionization modes. It was found out that flavonoids, tanshinones, and triterpenes had a stronger response in the positive ion mode compared to the negative ion mode. Nevertheless, the tested salvianolic acids showed sensitivity not only in positive mode but also in negative mode, rosmarinic acid, lithospermic acid, salvianolic acid B, and salvianolic acid A obtained from the negative ion mode were higher than that from the positive ion mode, while danshensu, protocatechuic aldehyde, and caffeic acid had better sensitivity and intensity in the positive ion mode, which made it accurate and easy to detect them with lower content levels in *S. miltiorrhiza* and identify each peak by confirming the molecular ions or quasi-molecular ions. Product ions were automatically chosen according to the stability and ion response by MS. The multiple reaction monitoring (MRM) transitions and parameters of 20 compounds applied in the study are listed in Table 1.

**Figure 2:**
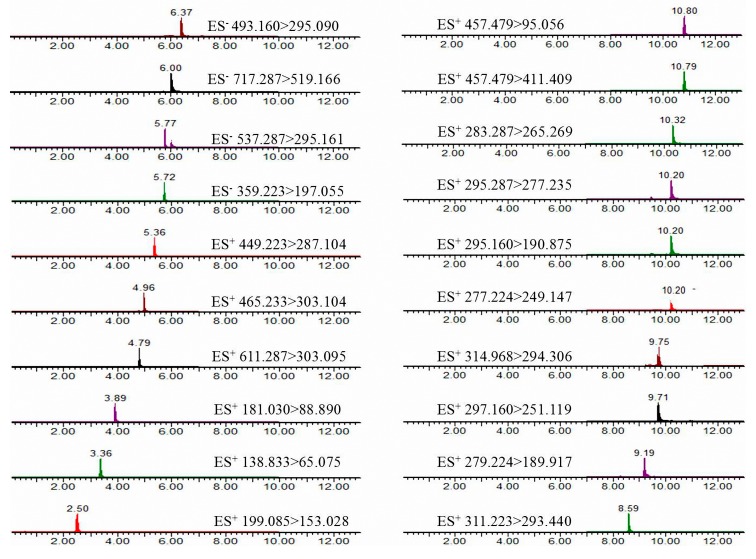
UPLC-TQ-MS/MS chromatogram of 20 compounds in *S. miltiorrhiza*.

**Figure 3:**
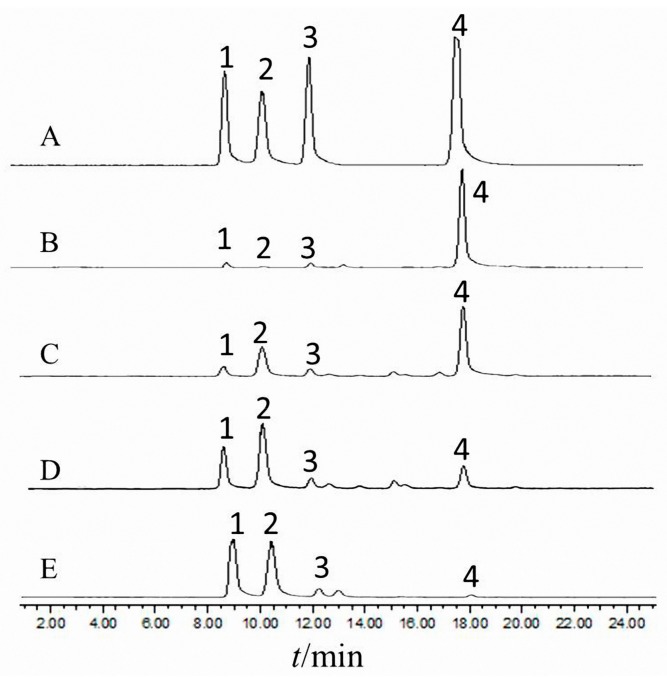
HPLC-ELSD chromatogram of different parts in *S. miltiorrhiza.* (**A**) Mixed reference; (**B**) roots; (**C**) stems; (**D**) leaves; and (**E**) flowers; (1) fructose; (2) glucose; (3) sucrose; (4); stachyose.

Chromatographic parameters were optimized for achieving a higher separation quality of the chromatogram and reducing the analysis time. As for the mobile phase, comparing with acetonitrile methanol, acetonitrile is a polar aprotic solvent that has been proven the best organic solvent for liquid chromatography, producing narrower peaks in a short analysis time, so that acetonitrile as the organic phase was optimal. In addition, different concentrations of formic acid added in ultrapure water as the aqueous phase was compared in our preliminary test and, finally, 0.1% formic acid in water/acetonitrile were tested for the good separation in determination. The typical chromatograms of 20 compounds are presented in Figure 2. Similarly, for the analysis of saccharides in *S. miltiorrhiza*, water/acetonitrile was chosen as the preferred mobile phase, and gradient elution was applied during the liquid chromatography process. The injection volume was set at 10 μL for samples of roots and leaves, while the injection volume was set to 20 μL for stems and flowers due to their lower contents. The flow rate was set at 0.8 mL/min and the column temperature was kept at 35 °C. Representative chromatograms of the reference standards are shown in Figure 3.

### 2.2. Method Validation

The developed UPLC-TQ-MS/MS method for quantitation of salvianolic acids, flavonoids, tanshinones, and triterpenes, and the HPLC-ELSD method for quantitation of saccharides were validated by determining the linearity, LODs, LOQs, precision, repeatability, stability, and recovery. The results are shown in Table 2. All of the marker substances showed good linearity with the determination coefficients (R^2^) ranging from 0.9900 to 0.9998 in a relatively wide concentration range. The results of precision test RSD values of the 24 analytes were less than 4.74%, repeatability and stability RSD values were less than 4.91%, and the overall recoveries were between 98.22% and 102.34% for the 24 reference compounds, with RSDs less than 3.61%. As a result, this showed that the established methods were accurate enough for the determination of 24 bioactive components in various parts of *S. miltiorrhiza* during different harvest times. Therefore, the UPLC-TQ-MS/MS and HPLC-ELSD methods are accurate, precise, and sensitive enough for quantitative evaluation of those salvianolic acids, flavonoids, tanshinones, triterpenes, and saccharides in the massive samples.

### 2.3. Distribution Characteristics of Five Kinds of Chemical Constituents in Different Parts of S. miltiorrhiza

The established UPLC-TQ-MS/MS method was well applied to the simultaneous determination of salvianolic acids (danshensu, protocatechuic aldehyde, caffeic acid, rosmarinic acid, lithospermic acid, salvianolic acid B, and salvianolic acid A), flavonoids (rutin, isoquercitrin, and astragalin), tanshinones (tanshinone IIB, dihydrotanshinone I, cryptotanshinone, neocryptotanshione, tanshinone I, tanshinone IIA, miltiradiene, and miltirone), and the triterpenes included oleanolic acid and ursolic acid. The results (Table 3) show that the main chemical components in roots of *S. miltiorrhiza* were salvianolic acids and tanshinones, without flavonoids and triterpenes being detected, while in the aerial parts of *S. miltiorrhiza*, it mainly contained the salvianolic acids, flavonoids, and triterpenes, and the liposoluble tanshinones were not detected. The results of this study are consistent with a previous report [18]. In general, the contents of total salvianolic acids in different parts of *S. miltiorrhiza* were: root > leaf > flower > stem; the contents of total flavonoids were leaf > flower > stem; and the triterpenes contents were flower > stem > leaf. Salvianolic acids were the common components of aerial parts and underground parts, and the concentration differs from each other at different growth stages. This prompts that salvianolic acids may be primarily biosynthesized in the leaves of *S. miltiorrhiza*, and meanwhile transferred to the roots by means of stems as the transport organ. It may also synthesize both in the aerial parts and underground parts and transported reciprocally [31].

The HPLC-ELSD method was applied for saccharide analysis of the samples, and the contents of the four saccharides (fructose, glucose, sucrose, and stachyose) are shown in Table 4, which revealed that the four saccharides exist in each part of *S. miltiorrhiza*, and the contents differ from each other. In the roots of *S. miltiorrhiza*, the content of stachyose, which possesses a high development value, was significantly higher than other parts of the plant, followed by the stems. Similarly, the disaccharide (sucrose) was detected mostly in the roots, as well; however, the two monosaccharides (fructose and glucose) mainly exist in leaves and flowers. The results revealed that in *S. miltiorrhiza*, the monosaccharides probably mainly synthetize in organs for photosynthesis, like leaves and flowers, while constituents of disaccharides and polysaccharides may form by dehydration with different kinds of monosaccharides in conducting tissue and vegetative organs, such as stems and roots [32]. Therefore, it can provide guidance for obtaining various saccharide resources from *S. miltiorrhiza*, as is known to us that saccharides and their derivatives may always be the important components that are responsible for the pharmacological activities of traditional Chinese medicine (TCM), for instance of stachyose, which has been confirmed for regulating gut microbiota and widely used for the development of health care products [33].

### 2.4. Dynamic Accumulation of Chemical Components in Roots of S. miltiorrhiza at Different Growth Stages

**Figure 4:**
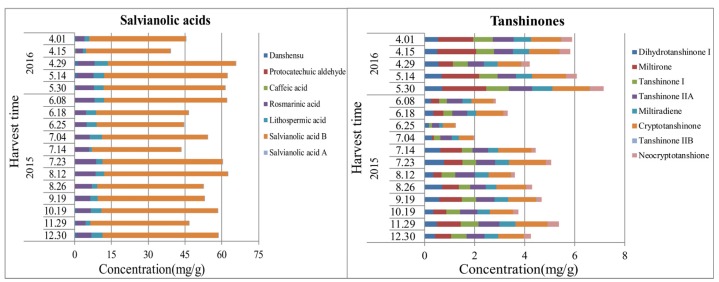
Dynamic accumulation of the contents of salvianolic acids and tanshinones in the roots of *S. miltiorrhiza*.

There is a certain growth correlation between cells, tissues, and organs in the plants: they are independent of each other and, meanwhile, closely related. In addition, the environmental factors, such as moisture, temperature, and light, show significant influence during plant growth and component accumulation [34]. Results (Figure 4 and Supplementary Materials Table S1) in the present study indicate that the content changing trend of salvianolic acids in roots of *S. miltiorrhiza* showed an “M” shape, as high as 6.598%; that is to say, the content of salvianolic acids reached the highest in the spring seedling stage (April to May) and the vigorous growth period of aerial parts (July to August). The results are in agreement with an earlier report [27]. With the temperature rising in spring, nutritive material in roots accumulate rapidly to make preparations for sprouting of the aerial parts. Afterwards, vegetative growth and reproductive growth of the aerial parts are in progress because of the high temperature and strong light in July and August, which results in strong photosynthesis in the leaves. As the aboveground parts and roots of plants form an organic combination which is inherently consistent, during the vigorous growth of aboveground parts, the effective components in roots were also accumulated at the highest levels in a year. As for the tanshinones (Table S2), they reached the highest level of 0.716% at the turning green stage of the next year. Considering the perennial plant as *S. miltiorrhiza*, content of tanshinones may accumulate in accordance with increased growth years. When it comes to the saccharides in *S. miltiorrhiza* of different growth periods, it contains a decent amount of stachyoses, and the content reached a maximum during the withering period. In general, two novel concerned active ingredients (salvianolic acids and tanshinones) enriched at the germination stage. Consequently, the optimum harvest time of roots from *S. miltiorrhiza* should be the seedling stage in spring.

### 2.5. Dynamic Accumulation of Chemical Components in the Aerial Parts of S. miltiorrhiza (Stems, Leaves, and Flowers) at Different Growth Stages

**Figure 5:**
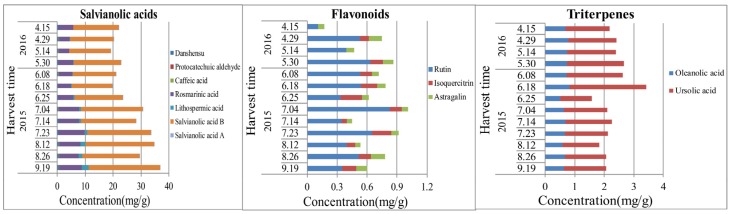
Dynamic accumulation of the contents of salvianolic acids, flavonoids, and triterpenes in the stems of *S. miltiorrhiza*.

As the results (Figure 5 and Table S3) indicate, salvianolic acids in the stems of *S. miltiorrhiza* increased in the whole growth period until mid- to late-September, at which time the aerial parts were tending to wither. However, the content of flavonoids and triterpenes reached the highest levels in mid-June and early July, respectively, probably because of the key enzymes that are involved in the synthesis of flavonoids and triterpenes are at the highest content levels and activity in this period [35]. The contents of monosaccharides (fructose and glucose) showed the highest values in wilting and seedling stages, and disaccharide (sucrose) and oligosaccharide (stachyose) reached their maxima in the vigorous growth period of the aerial parts in July. Further studies are needed to clarify the dynamic accumulation of saccharides in stems.

**Figure 6:**
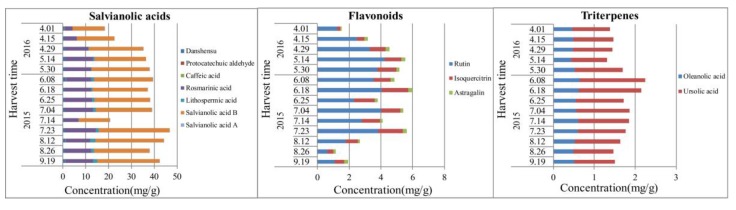
Dynamic accumulation of the contents of salvianolic acids, flavonoids, and triterpenes in the leaves of *S. miltiorrhiza*.

During the whole growth period of the plant, concentrations of salvianolic acids, flavonoids, and triterpenes in leaves increased firstly, and declined afterwards, for the duration of late July to early August, when the temperature and illumination were the highest. For leaves as the synthetic organ, the content of salvianolic acids reached the maximal value, while the maximum accumulation of flavonoids and triterpenes occurs in June. The results are shown in Figure 6 and Table S4. Similarly with the stems, the disaccharide (sucrose) and oligosaccharide (stachyose) contents reached the highest values in July, at which time the photosynthesis was the strongest. Moreover, monosaccharides (fructose and glucose) accumulated to the maximum level in May.

**Figure 7:**
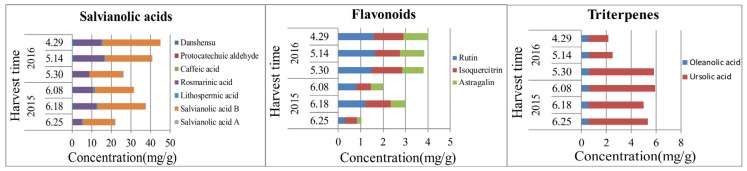
Dynamic accumulation of the contents of salvianolic acids, flavonoids, and triterpenes in the flowers of *S. miltiorrhiza*.

Analysis of the dynamic accumulation of chemical components in flowers of *S. miltiorrhiza* at different flowering stages is shown in Figure 7 and Table S5 indicates that the contents of phenolic acids and flavonoids reached the maximum value at the end of April (early bloom stage), while the triterpenes and monosaccharides (fructose and glucose) were high at the end of May to early June (full-bloom stage), and the disaccharide (sucrose) and oligosaccharide (stachyose) contents attained the highest levels at the middle to the end of June (late bloom stage). From what is mentioned above, we may come to the conclusion that chemical components of the aerial parts of *S. miltiorrhiza* were various and abundant, which can be taken into consideration as a new source of salvianolic acids and flavonoids. As a matter of fact, determination of the suitable harvest season of the aerial parts of *S. miltiorrhiza* should be evaluated according to the actual demand and, supposing salvianolic acid as the main evaluation index, we recommend July and August as the optimum harvest time of the aerial parts of *S. miltiorrhiza*.

## 3. Materials and Methods

### 3.1. Chemicals and Reagents

Acetonitrile and formic acid (HPLC grade) were all purchased from Merck (Darmstadt, Germany); deionized water was obtained by a Milli-Q water purification system (Millipore, Billerica, MA, USA). Other chemicals and reagents were of analytical grade. Chemical standards, including protocatechuic aldehyde (2), caffeic acid (3), rosmarinic acid (7), rutin (4), tanshinone IIA (16), and cryptotanshinone (13), were purchased from the National Institute for the Control of Pharmaceutical and Biological Products. Danshensu (1), lithospermic acid (8), salvianolic acid B (9), salvianolic acid A (10), isoquercitrin (5), astragalin (6), dihydrotanshinone I (12), and tanshinone I (15) were purchased from Putian Tongchuang Biotechnology Co. Ltd. (Beijing, China). Tanshinone IIB (11), neocryptotanshione (14), miltiradiene (17), and miltirone (18) were purchased from Guyan Industrial Co., Ltd. (Shanghai, China). Oleanolic acid (19) and ursolic acid (20) were purchased from Preferred Biological Technology Co., Ltd. (Chengdu, China). Fructose (21), glucose (22), and sucrose (23) were purchased from Spring and Autumn Biological Engineering Co., Ltd. (Nanjing, China), and the stachyose (24) was purchased from Chroma-Biotechnology Co., Ltd. (Chengdu, China). The purity of each compound was over 98%, as determined by HPLC analysis.

### 3.2. Plant Materials

The samples were collected from the medicinal botanical garden of Nanjing University of Chinese Medicine between June 2015 to May 2016, and their botanical origins were identified by Prof. Jin-ao Duan of the Jiangsu Collaborative Innovation Center of Chinese Medicinal Resources Industrialization, Nanjing University of Chinese Medicine, Nanjing, China. The samples were separated into different parts as roots, leaves, stems, and flowers after collection, dried at 50 °C individually, then pulverized into homogeneous powders (40 mesh) and stored dry at room temperature before analysis. The voucher specimens were deposited in the Herbarium of the Nanjing University of Chinese Medicine. Information about these samples is summarized in Table 5.

### 3.3. Preparation of Standard Solutions

#### 3.3.1. Standard Solution Preparation of Salvianolic Acids, Flavonoids, Tanshinones, and Triterpenes

A mixed standard stock solution containing the reference compounds **1**–**20** was prepared in 90% methanol, and the concentration for the 20 analytes were as follows: 0.229, 0.162, 0.190, 0.202, 0.185, 0.148, 0.253, 0.190, 0.257, 0.174, 0.071, 0.126, 0.236, 0.186, 0.107, 0.113, 0.089, 0.144, 0.176, and 0.207 mg/mL. Working standard solutions for calibration curves were prepared by diluting the mixed standard stock solution with 80% methanol to different concentrations.

#### 3.3.2. Standard Solution Preparation of Saccharides

The mixed standard stock solution containing the reference compounds **21**–**24** was prepared with Millipore water at concentrations of 3.20, 3.05, 2.61, and 5.03 mg/mL, then diluted to different concentrations. All of the solutions were stored in a refrigerator at 4 °C until use and filtered through a 0.22 μm cellulose membrane before analysis.

### 3.4. Preparation of Sample Solutions

#### 3.4.1. Sample Solutions Preparation for Salvianolic Acids, Flavonoids, Tanshinones, and Triterpenes Analysis

The dried powder (0.5 g) from various parts of *S. miltiorrhiza* during different harvest times, which was weighed accurately, was put into a 50 mL conical flask with a stopper, and 50 mL 80% methanol was added. After accurate weighing, ultrasonication (100 kHz) was performed at 50 °C for 45 min; afterwards the same solvent was added to compensate for the weight lost during extraction.

#### 3.4.2. Sample Solutions Preparation for Saccharides Analysis

As above, the dried powder (0.5 g) was weighed accurately and 50 mL ultrapure water was added. After accurate weighing, reflux extraction was performed for 1 h and compensation for the weight lost with ultrapure water was conducted. After centrifugation (13,000 rpm, 10 min) and filtering through a 0.22 μm membrane filter, all of the sample solutions were stored at 4 °C before the injection into UPLC-TQ-MS/MS and HPLC-ELSD systems for analysis.

### 3.5. Chromatographic Conditions and Instrumentation

#### 3.5.1. Analysis for Salvianolic Acids, Flavonoids, Tanshinones, and Triterpenes.

Analysis was performed on a Waters Acquity UPLC system (Waters, Milford, MA, USA), which consists of a quaternary pump solvent management system, an online degasser, an autosampler, and a triple quadrupole mass detector. An Acquity UPLC BEH C_18_ (2.1 mm × 100 mm, 1.7 μm) column was applied for all analyses. The mobile phase was composed of A (water and 0.1% formic acid) and B (acetonitrile) using a gradient elution of 3% B at 0–1 min, 3–30% B at 1–6 min, 30–40% B at 6–7 min, 40–95% B at 7–10 min, and 95–95% B at 10–12 min. The flow rate of the mobile phase was set at 0.40 mL/min. The column temperature was conditioned at 35 °C, the autosampler was maintained at 4 °C, and the injection volume was 2 μL.

The triple quadrupole (TQ) mass spectrometer was operated in both positive and negative modes with a capillary voltage of 3 kV, a cone gas flow of 20 L/h, a collision gas flow of 0.15 mL/min, a desolvation gas flow of 1000 L/h, a desolvation temperature of 550 °C, a source temperature of 150 °C, and full-scan spectra from 100 to 1000 Da. The raw data were acquired and processed with MassLynx 4.1 software (Waters Corporation, Milford, MA, USA).

#### 3.5.2. Analysis for Saccharides

The HPLC-ELSD conditions were used for the determination of the saccharides, a Waters Alliance 2695 liquid chromatograph system (Waters, Milford, MA, USA) equipped with an Alltech 2000 evaporative light-scattering detector (Grace, Deerfield, MA, USA) was used. The chromatographic separations were performed over a Prevail Carbohydrate ES (250 mm × 4.6 mm, 5 μm) column at a column temperature of 35 °C. The column was eluted with a mixture of acetonitrile (mobile phase A) and water (mobile phase B) at a flow rate of 1.0 mL/min. The elution conditions were as follows: 0–23 min, 20–50% B; 23–26 min, and 50–20% B. The drift tube temperature of the ELSD was set at 80 °C, and using nitrogen as the carrier gas at a flow rate set at 3.0 L/min, the gain value was 10, and the injection volume was 10 μL for roots and leaves analysis, and 20 μL for stems and flowers analysis.

### 3.6. Validation of the Methods

#### 3.6.1. Calibration Curves, LODs, and LOQs

The linearity was obtained by preparing a series of concentrations of standard solution with at least five appropriate concentrations in duplicate. The lowest concentration of the working solution for calibration use was diluted with the corresponding solvent to a series of concentrations. LODs and LOQs for each analyte were acquired while the S/N was 3 and 10, respectively.

#### 3.6.2. Precision, Repeatability, and Stability

To evaluate the precision, we analyzed the standard solutions with six replicates, and the RSD of the peak area for each standard compounds was calculated. To confirm the repeatability in this developed method, six different sample solutions were prepared from the same sample (sample Y1, the leaves of SM which were collected on 8 June 2015) were analyzed and variations were expressed by RSD. The stability was evaluated by storing the sample solutions mentioned above (Y1) at 25 °C, then analyzed at 0, 2, 4, 8, 12, and 24 h, respectively.

#### 3.6.3. Recovery

A spike recovery test was used to evaluate the accuracy of these methods, which was performed by adding the corresponding compounds at high (120%), medium (100%), and low (80%) levels in the sample (Y1) preparation, then measured in six duplicates. The spiked samples were then extracted, processed, and quantified in accordance with the methods mentioned above. The spike recoveries were calculated using the following equation:

Recovery % = [(measured amount − original amount)/amount added] × 100%.

### 3.7. Sample Determination

All of the samples were prepared according to Section 3.4, and determined thereafter according to the Section 3.5 chromatographic conditions and instrumentation. Quantification of each compound was performed on the basis of linear calibration plots of the peak areas versus the corresponding concentration. The content of total phenolic acids was the sum of danshensu, protocatechuic aldehyde, caffeic acid, rosmarinic acid, lithospermic acid, salvianolic acid B, and salvianolic acid A. The sum of rutin, isoquercitrin, and astragalin defined the total flavonoids. Total tanshinones was defined as the sum of tanshinone IIB, dihydrotanshinone I, cryptotanshinone, neocryptotanshione, tanshinone I, tanshinone IIA, miltiradiene and miltirone. The total triterpenes included oleanolic acid and ursolic acid.

## 4. Conclusions

In the present study, the simple and reliable UPLC-TQ-MS/MS and HPLC-ELSD analytical methods for rapidly quantifying multiple components in *S. miltiorrhiza* was established. As a result, 24 components were identified and quantified, which clearly suggested the aerial parts of *S. miltiorrhiza* contain phenolic acids, flavonoids, triterpenes, and saccharides, without the liposoluble tanshinones being detected. Therefore, the aerial parts of *S. miltiorrhiza* could be a promising natural source for phenolic acids and flavonoids. During the planting production of TCM, suitable harvest time is crucial for the yield and quality of medicinal materials. To the authors’ knowledge, this is the first report about distribution and dynamic changes of chemical constituents in various parts of *S. miltiorrhiza* during different harvest seasons. Apart from the difference of chemical composition between aboveground and underground parts of *S. miltiorrhiza*, the data provided an effective reference for the optimal harvest time selection according to the accumulation dynamics of target components. It recommended that the optimal harvest time for the roots and rhizomes of *S. miltiorrhiza* is the seedling stage in spring and, for the aerial parts of *S. miltiorrhiza*, is July to August. The research could provide the theoretical basis and scientific evidence for comprehensive development and utilization of *S. miltiorrhiza* resources.

Since all of the samples were collected from the medicinal botanical garden of Nanjing University of Chinese Medicine, dynamic accumulation of chemical constituents in various parts of *S. miltiorrhiza* during different growth periods from other geographical areas is unclear, which remains of interest for further research. Nevertheless, the results and established methods of this paper were intended to provide a favorable reference for the study of *S. miltiorrhiza* from other origins. Furthermore, the established UPLC-TQ-MS/MS and HPLC-ELSD methods can also be applied in future research of *S. miltiorrhiza* and its preparation of medicines.

## Figures and Tables

**Table 1:** MS/MS detection parameters of 20 compounds in *S. miltiorrhiza*.

Compounds	*t*_R_ (min)	MW	MRM Transitions/SIM	Cone Voltage (V)	Collision Energy (eV)	Ion Mode
1. Danshensu	2.50	198.05	199.085 > 153.028	10	6	ES^+^
2. Protocatechuic aldehyde	3.36	138.12	138.833 > 65.075	16	20	ES^+^
3. Caffeic acid	3.89	180.04	181.030 > 88.890	12	26	ES^+^
4. Rutin	4.79	610.51	611.287 > 303.095	14	22	ES^+^
5. Isoquercitrin	4.96	464.38	465.233 > 303.104	32	22	ES^+^
6. Astragalin	5.36	448.00	449.223 > 287.104	30	22	ES^+^
7. Rosmarinic acid	5.72	360.31	359.223 > 197.055	30	16	ES^−^
8. Lithospermic acid	5.77	538.46	537.287 > 295.161	36	18	ES^−^
9. Salvianolic acid B	6.00	718.62	717.287 > 519.166	26	18	ES^−^
10. Salvianolic acid A	6.37	494.12	493.16 > 295.09	28	18	ES^−^
11. Tanshinone IIB	8.60	310.00	311.223 > 293.44	22	12	ES^+^
12.Dihydrotanshinone I	9.22	278.30	279.224 > 189.917	28	34	ES^+^
13. Cryptotanshinone	9.71	296.35	297.16 > 251.119	36	26	ES^+^
14. Neocryptotanshione	9.75	314.38	314.968 > 294.306	86	6	ES^+^
15. Tanshinone I	10.20	276.29	277.224 > 249.147	30	20	ES^+^
16.Tanshinone IIA	10.20	294.34	295.16 > 190.875	32	44	ES^+^
17. Miltiradiene	10.20	272.47	295.287 > 277.235	28	20	ES^+^
18. Miltirone	10.32	282.38	283.287 > 265.269	30	18	ES^+^
19. Oleanolic acid	10.80	456.71	457.479 > 411.409	12	16	ES^+^
20. Ursolic acid	10.82	456.68	457.479 > 95.056	14	30	ES^+^

**Table 2:** Calibration curves, LOD, LOQ, precision, repeatability, stability, and recovery of the 24 references.

Compounds	Calibration Curves	R^2^	Linear Range/μg/mL	LOD/ng/mL	LOQ/ng/mL	Precision (%, *n* = 6)	Repeatability (%, *n* = 6)	Stability (%, *n* = 6)	Recovery (%, *n* = 3)	
									Mean	RSD
1. Danshensu	*Y* = 1449.8*X* − 477.58	0.9998	1.15~114.50	55.91	186.36	3.13	3.24	4.26	101.07	2.13
2.Protocatechuic aldehyde	*Y* = 23160*X* − 1692.6	0.9982	0.16~16.20	7.63	25.42	3.12	4.38	3.31	99.65	3.21
3. Caffeic acid	*Y* = 9923.3*X* − 221.72	0.9981	0.19~19.00	14.55	48.50	3.33	3.72	3.36	99.86	2.21
4. Rutin	*Y* = 8337.1*X* + 17508	0.9986	1.01~101.00	4.05	13.50	2.97	3.59	4.29	100.79	2.43
5. Isoquercitrin	*Y* = 6526.3*X* + 7495.6	0.9996	0.93~92.50	7.05	23.50	1.12	2.87	3.62	99.09	1.99
6. Astragalin	*Y* = 18160*X* − 3328.2	0.9994	0.74~74.00	4.09	13.64	2.11	4.39	2.42	101.91	3.21
7. Rosmarinic acid	*Y* = 2132.8*X* + 4654.1	0.9989	1.27~126.50	22.85	76.16	1.68	3.96	1.82	98.68	2.65
8. Lithospermic acid	*Y* = 375.37*X* + 1307.5	0.9915	1.90~380.00	93.37	311.24	2.10	4.10	3.64	99.76	3.21
9. Salvianolic acid B	*Y* = 1292.2*X* + 4951.3	0.9986	1.29~257.00	66.37	221.22	1.73	3.85	1.35	101.25	1.34
10. Salvianolic acid A	*Y* = 4008.9*X* + 172.38	0.9972	0.174~17.40	33.08	110.27	1.87	3.90	3.36	99.82	2.93
11. Tanshinone IIB	*Y* = 133524*X* + 35,802	0.9967	0.07~7.10	3.12	10.40	1.73	3.21	4.91	102.23	2.36
12.Dihydrotanshinone I	*Y* = 232666*X* + 126,802	0.9972	0.13~12.60	8.20	27.33	2.86	4.55	2.43	98.22	3.25
13.Cryptotanshinone	*Y* = 293851*X* + 578,254	0.9955	0.24~23.60	11.42	38.06	4.55	4.91	2.78	101.15	1.52
14.Neocryptotanshione	*Y* = 100.92*X* + 24.255	0.9976	0.19~18.60	213.67	712.29	2.86	3.15	3.12	100.18	2.83
15. Tanshinone I	*Y* = 32735*X* + 19,739	0.9961	0.11~10.70	40.04	133.48	2.25	3.48	3.47	99.82	3.29
16.Tanshinone IIA	*Y* = 355880*X* + 261,945	0.9942	0.113~11.30	5.24	17.47	4.74	1.19	4.26	102.34	1.58
17. Miltiradiene	*Y* = 794169*X*+ 561,247	0.9954	0.09~8.90	2.73	9.10	4.60	4.06	4.28	100.34	3.28
18. Miltirone	*Y* = 88227*X* + 109,759	0.9991	0.72~72.00	4.53	15.11	1.14	3.94	4.08	101.64	2.43
19. Oleanolic acid	*Y* = 8484.5*X* + 6510.4	0.9988	0.18~17.60	14.93	49.78	3.01	4.02	3.73	99.01	1.54
20. Ursolic acid	*Y* = 1064.6*X* + 5087.3	0.9979	1.04~103.50	38.37	129.91	2.57	4.44	4.15	99.90	3.24
21. Fructose	*Y* = 1.4383*X* + 5.879	0.9923	32~3200	4.14 × 10^4^	1.38 × 10^5^	1.34	2.78	3.11	100.35	2.45
22. Glucose	*Y* = 1.3669*X* + 6.6307	0.9937	30.54~3054.00	2.52 × 10^4^	8.39 × 10^4^	1.83	2.69	2.13	101.35	2.52
23. Sucrose	*Y* = 1.2227*X* + 7.9658	0.9941	26.06~2606.00	3.83 × 10^4^	1.28 × 10^5^	1.75	3.88	2.47	102.23	3.61
24. Stachyose	*Y* = 1.2012*X* + 7.9475	0.9900	50.26~5026.00	2.19 × 10^4^	7.31 × 10^4^	1.92	2.31	3.23	99.43	1.35

**Table 3:** Contents of salvianolic acids, tanshinones, flavonoids, and triterpenes in different growth periods of different parts in *S.miltiorrhiza* (mg/g).

Harvest Year	Harvest Date	Total Phenolic Acids				Total Tanshinones	Total Flavonoids			Total Triterpenes		
		Roots	Stems	Leaves	Flowers	Roots	Stems	Leaves	Flowers	Stems	Leaves	Flowers
2016	4.01	45.30	n	18.25	n	5.90	n	1.53	n	n	1.38	n
	4.15	39.17	22.05	22.66	n	5.81	0.17	3.17	n	2.17	1.47	n
	4.29	65.98	20.30	35.24	44.96	4.21	0.75	4.54	4.02	2.42	1.45	2.15
	5.14	62.23	19.25	36.40	40.81	6.09	0.47	5.53	3.85	2.39	1.31	2.52
	5.30	61.42	22.80	37.90	25.99	7.16	0.86	5.16	3.82	2.66	1.70	5.82
2015	6.08	62.11	21.17	39.49	31.36	2.85	0.71	4.84	2.00	2.63	2.24	5.91
	6.18	46.61	19.76	37.00	37.42	3.32	0.78	5.97	2.98	3.43	2.15	5.00
	6.25	44.60	23.55	38.16	21.97	1.24	0.62	3.79	1.05	1.57	1.72	5.33
	7.04	54.29	30.58	38.81	n	1.99	1.01	5.40	n	2.11	1.87	n
	7.14	43.48	28.22	20.53	n	4.44	0.45	4.11	n	2.25	1.85	n
	7.23	60.33	33.57	46.68	n	5.06	0.92	5.63	n	2.12	1.77	n
	8.12	62.45	34.74	44.14	n	3.61	0.54	2.68	n	1.84	1.64	n
	8.26	52.59	29.58	37.91	n	4.29	0.78	1.15	n	2.06	1.47	n
	9.19	52.91	36.82	42.25	n	4.68	0.60	1.93	n	2.06	1.51	n
	10.19	58.34	n	n	n	3.74	n	n	n	n	n	n
	11.29	46.69	n	n	n	5.37	n	n	n	n	n	n
	12.30	58.73	n	n	n	4.26	n	n	n	n	n	n

The “n” represents without harvest.

**Table 4:** Contents of saccharides in different growth periods of different parts in *S. miltiorrhiza* (mg/g).

Harvest Year	Harvest Date	Glucose				Sucrose				Stachyose			
		Roots	Stems	Leaves	Flowers	Roots	Stems	Leaves	Flowers	Roots	Stems	Leaves	Flowers
2016	4.01	-	n	11.10	n	3.06	n	-	n	229.86	n	1.62	n
	4.15	2.40	41.51	19.51	n	2.08	1.23	-	n	131.03	3.79	2.91	n
	4.29	-	31.43	46.06	41.30	7.19	1.60	2.01	4.50	159.14	2.03	5.55	0.81
	5.14	-	9.34	46.23	52.03	3.98	0.68	2.28	1.58	129.43	3.32	4.72	0.49
	5.30	-	9.12	40.68	17.77	2.22	-	1.28	0.51	109.72	4.61	3.35	1.15
2015	6.08	5.75	22.62	29.50	27.03	11.54	0.53	0.78	1.15	279.50	22.05	-	2.08
	6.18	-	4.79	23.80	42.92	5.21	0.70	1.61	5.07	244.40	4.20	1.28	2.16
	6.25	1.96	15.83	10.63	8.99	6.38	2.44	1.17	-	175.97	6.38	3.81	6.69
	7.04	3.69	20.31	35.48	n	14.47	3.06	4.46	n	268.33	27.69	1.85	n
	7.14	1.74	8.64	10.53	n	7.41	2.41	2.42	n	269.66	29.24	-	n
	7.23	-	12.77	9.62	n	7.62	2.58	2.29	n	286.16	33.07	2.03	n
	8.12	-	11.60	5.39	n	8.63	1.73	0.76	n	253.15	23.93	1.61	n
	8.26	3.37	10.88	13.98	n	3.73	1.35	2.10	n	173.98	3.64	1.22	n
	9.19	4.79	12.40	23.64	n	9.84	1.26	1.07	n	239.77	10.98	0.98	n
	10.19	2.38	n	n	n	15.41	n	n	n	297.15	n	n	n
	11.29	-	n	n	n	11.67	n	n	n	299.71	n	n	n
	12.30	3.70	n	n	n	22.70	n	n	n	275.49	n	n	n

The “-” represents not detected and “n” represents without harvest.

**Table 5:** Harvest time, growth period, and harvest parts of *S. miltiorrhiza*.

Collecting Time (Day Month Year)	Growth Stage	Phenological Phase	Harvest Parts
1 April 2016	Turn green	Leaf expansion period	Roots, Leaves
15 April 2016			Roots, Stems, Leaves
29 April 2016	Vegetative growth and reproductive growth of the aerial parts	Early bloom stage	Roots, Stems, Leaves, Flowers
14 May 2016			Roots, Stems, Leaves, Flowers
30 May 2016		Full-bloom stage	Roots, Stems, Leaves, Flowers
8 June 2015			Roots, Stems, Leaves, Flowers
18 June 2015		Late bloom stage	Roots, Stems, Leaves, Flowers
25 June 2015			Roots, Stems, Leaves, Flowers
4 July 2016		Branch growth period	Roots, Stems, Leaves
14 July 2015			Roots, Stems, Leaves
23 July 2015			Roots, Stems, Leaves
12 August 2015		Form stable phase of the aerial parts	Roots, Stems, Leaves
26 August 2015			Roots, Stems, Leaves
19 September 2015	Vegetative growth of the roots	Rapid growing stage of roots	Roots, Stems, Leaves
19 October 2015	As above	Late rapid growing stage of roots	Roots
29 November 2015	Growth arrest	Withering stage	Roots
30 December 2015	Growth arrest	Dormancy period	Roots

## Supplementary Materials

Supplementary materials are available online.
