# Supplementary material for: Comparative Analysis of the Major Chemical Constituents in Salvia miltiorrhiza Roots, Stems, Leaves and Flowers during Different Growth Periods by UPLC-TQ-MS/MS and HPLC-ELSD Methods
Source: Molecules. 2017 May 10;22(5):771. doi: 10.3390/molecules22050771 (PMC6154317; doi:10.3390/molecules22050771)
Supplement: Supplementary file 1 [file molecules-22-00771-s001.pdf]

Table S1. Dynamic accumulation on the contents of salvianolic acids in roots of *S.miltiorrhiza* (mg/g)

Harvest year	Harvest date	Danshensu	Protocatechuic aldehyde	Caffeic acid	Rosmarinic acid	Lithospermic acid	Salvianolic acid B	Salvianolic acid A
2016	4.01	0.513	0.020	0.039	3.434	1.848	39.403	0.033
	4.15	0.370	0.019	0.040	2.823	1.308	34.584	0.016
	4.29	1.353	0.018	0.039	6.636	5.507	52.174	0.199
	5.14	0.951	0.022	0.044	6.638	4.231	50.290	0.027
	5.30	0.836	0.022	0.033	7.085	3.741	49.642	0.040
2015	6.08	0.052	0.008	0.007	7.932	3.971	50.020	0.080
	6.18	0.115	0.008	0.006	4.548	4.036	37.768	0.078
	6.25	0.178	0.008	0.006	4.613	4.240	35.384	0.112
	7.04	0.133	0.009	0.010	6.101	4.794	43.110	0.093
	7.14	0.075	0.009	0.012	5.840	1.139	36.377	0.027
	7.23	0.113	0.010	0.014	8.523	2.738	48.883	0.043
	8.12	0.229	0.010	0.013	8.206	3.589	50.333	0.061
	8.26	0.189	0.013	0.028	6.799	2.238	43.267	0.044
	9.19	0.313	0.014	0.025	5.941	3.035	43.494	0.068
	10.19	0.701	0.011	0.017	5.911	4.330	47.258	0.081
	11.29	0.470	0.016	0.030	3.819	2.126	40.070	0.137
	12.30	0.898	0.013	0.023	5.932	4.379	47.379	0.075

Table S2. Dynamic accumulation on the contents of tanshinones in roots of *S.miltiorrhiza* (mg/g)

Harvest year	Harvest date	Dihydrotanshinone I	Miltirone	Tanshinone I	Tanshinone IIA	Miltiradiene	Cryptotanshinone	Tanshinone IIB	Neocryptotanshione
2016	4.01	0.546	1.406	0.764	0.843	0.691	1.220	0.014	0.417
	4.15	0.520	1.542	0.715	0.767	0.626	1.230	0.015	0.398
	4.29	0.557	0.563	0.611	0.649	0.544	0.951	0.011	0.324
	5.14	0.676	1.514	0.727	0.749	0.637	1.345	0.015	0.430
	5.30	0.715	1.750	0.894	0.941	0.802	1.509	0.020	0.528
2015	6.08	0.247	0.344	0.309	0.612	0.356	0.880	0.001	0.101
	6.18	0.346	0.410	0.346	0.598	0.364	1.083	0.002	0.168
	6.25	0.186	-	0.119	0.283	0.132	0.491	-	0.030
	7.04	0.293	0.070	0.268	0.460	0.274	0.581	0.001	0.046
	7.14	0.621	0.873	0.413	0.624	0.415	1.323	0.003	0.167
	7.23	0.775	0.750	0.540	0.755	0.539	1.484	0.008	0.214
	8.12	0.342	0.345	0.544	0.780	0.542	0.920	0.006	0.137
	8.26	0.714	0.652	0.457	0.608	0.434	1.188	0.007	0.232
	9.19	0.577	0.917	0.569	0.735	0.543	1.123	0.011	0.202
	10.19	0.350	0.509	0.558	0.689	0.510	0.917	0.006	0.203
	11.29	0.491	0.960	0.708	0.837	0.644	1.267	0.012	0.451
	12.30	0.421	0.648	0.620	0.698	0.553	1.016	0.008	0.292

The "-" represents not detected.

Table S3. Dynamic accumulation on the contents of salvianolic acids, flavonoids, and triterpenes in the stems of *S. miltiorrhiza* (mg/g)

Harvest year	Harvest date	Danshensu	Protocatechuic aldehyde	Caffeic acid	Rosmarinic acid	Lithospermic acid	Salvianolic acid B	Salvianolic acid A	Rutin	Isoquercitrin	Astragalin	Oleanolic acid	Ursolic acid
2016	4.15	0.486	0.022	0.083	5.088	0.057	16.298	0.007	0.108	-	0.066	0.693	1.481
	4.29	0.539	0.041	0.074	3.852	0.059	15.600	0.063	0.530	0.085	0.133	0.776	1.640
	5.14	0.419	0.017	0.050	3.816	-	14.942	-	0.392	0.001	0.078	0.745	1.649
	5.30	0.366	0.018	0.063	5.354	0.071	16.913	0.010	0.635	0.127	0.102	0.750	1.915
2015	6.08	0.391	0.035	0.079	4.979	0.120	15.434	0.064	-	-	0.067	0.724	1.901
	6.18	0.355	0.022	0.053	4.574	0.166	14.513	0.049	0.538	0.162	0.085	0.817	2.611
	6.25	0.320	0.020	0.058	5.397	0.438	17.268	0.028	0.337	0.213	0.068	0.512	1.061
	7.04	0.397	0.020	0.069	7.309	0.762	21.991	0.026	0.826	0.121	0.063	0.633	1.478
	7.14	0.487	0.023	0.076	7.216	0.657	19.702	0.046	0.345	0.054	0.051	0.691	1.562
	7.23	0.477	0.025	0.082	9.175	0.998	22.742	0.060	0.649	0.192	0.075	0.679	1.438
	8.12	0.534	0.028	0.097	7.629	1.901	24.513	0.034	0.396	0.087	0.052	0.585	1.253
	8.26	0.509	0.077	0.122	7.013	1.339	20.360	0.115	0.516	0.124	0.142	0.664	1.396
	9.19	0.540	0.024	0.081	8.224	2.361	25.533	0.048	0.351	0.139	0.111	0.634	1.424

The "-" represents not detected.

Table S4. Dynamic accumulation on the contents of salvianolic acids, flavonoids, and triterpenes in the leaves of *S. miltiorrhiza* (mg/g)

Harvest year	Harvest date	Danshensu	Protocatechuic aldehyde	Caffeic acid	Rosmarinic acid	Lithospermic acid	Salvianolic acid B	Salvianolic acid A	Rutin	Isoquercitrin	Astragalin	Oleanolic acid	Ursolic acid
2016	4.01	0.765	0.021	0.074	3.420	-	13.960	0.009	1.246	0.188	0.097	0.461	0.923
	4.15	0.677	0.020	0.083	5.360	-	16.515	-	2.483	0.469	0.214	0.487	0.985
	4.29	1.011	0.024	0.075	10.075	0.224	23.823	0.012	3.310	0.995	0.233	0.472	0.976
	5.14	1.070	0.023	0.095	12.090	0.466	22.651	-	4.235	1.057	0.241	0.437	0.874
	5.30	0.814	0.025	0.102	11.382	0.356	25.215	0.008	3.766	1.206	0.193	0.522	1.177
2015	6.08	1.241	0.086	0.160	10.924	1.284	25.687	0.106	3.562	1.034	0.248	0.643	1.598
	6.18	1.219	0.026	0.095	10.991	0.679	23.967	0.020	4.035	1.689	0.251	0.619	1.534
	6.25	1.000	0.036	0.136	11.604	0.992	24.383	0.010	2.313	1.283	0.191	0.551	1.173
	7.04	0.876	0.023	0.080	12.268	1.313	24.245	0.007	4.024	1.189	0.191	0.562	1.305
	7.14	0.606	0.027	0.075	5.953	0.197	13.641	0.027	2.801	1.164	0.147	0.603	1.245
	7.23	1.059	0.026	0.103	13.379	1.248	30.826	0.033	3.813	1.560	0.259	0.591	1.182
	8.12	1.155	0.036	0.126	10.560	2.333	29.914	0.016	1.785	0.768	0.130	0.524	1.116
	8.26	0.859	0.028	0.080	11.350	1.368	24.210	0.011	0.633	0.374	0.140	0.493	0.979
	9.19	0.878	0.029	0.108	12.278	1.793	27.140	0.022	1.085	0.597	0.249	0.509	1.000

The "-" represents not detected.

Table S5. Dynamic accumulation on the contents of salvianolic acids, flavonoids, and triterpenes in the flowers of *S. miltiorrhiza* (mg/g)

Harvest year	Harvest date	Danshensu	Protocatechuic aldehyde	Caffeic acid	Rosmarinic acid	Lithospermic acid	Salvianolic acid B	Salvianolic acid A	Rutin	Isoquercitrin	Astragalin	Oleanolic acid	Ursolic acid
2016	4.29	1.304	0.021	0.123	13.555	0.606	29.075	0.274	1.595	1.313	1.115	0.567	1.587
	5.14	0.979	0.033	0.223	15.148	0.480	23.656	0.294	1.638	1.104	1.108	0.592	1.924
	5.30	0.664	0.022	0.091	8.163	-	16.978	0.072	1.490	1.364	0.968	0.582	5.234
2015	6.08	0.796	0.023	0.109	9.820	0.789	19.756	0.069	0.812	0.644	0.544	0.596	5.312
	6.18	0.792	0.029	0.139	11.344	0.760	24.248	0.111	1.225	1.114	0.638	0.550	4.447
	6.25	0.329	0.027	0.062	4.576	0.430	16.514	0.028	0.289	0.540	0.220	0.555	4.780

The "-" represents not detected.
